# Supplementary material for: CDH2 mutation affecting N-cadherin function causes attention-deficit hyperactivity disorder in humans and mice
Source: Nat Commun. 2021 Oct 26;12:6187. doi: 10.1038/s41467-021-26426-1 (PMC8548587; doi:10.1038/s41467-021-26426-1)
Supplement: Supplementary file 1 — Supplementary Information [file 41467_2021_26426_MOESM1_ESM.pdf]

Supplementary

This file includes:

- Supplementary Table 1 - GO Term functional analysis of differentially expressed genes
- Supplementary Table 2 - GO Biological process analysis – ventral midbrain (vMB)
- Supplementary Table 3 - GO Biological processing analysis – prefrontal cortex (PFC)
- Supplementary Table 4 - Differentially expressed genes in *Cdh2* knock-in mice
- Supplementary Table 5 - Primers and oligos
- Supplementary Figure 1 - Mass spectra data for LC-MS experiments
- Supplementary Figure 2 - Knock-in mice phenotypic evaluation and histological assessment

Supplementary Tables 1

GO Term functional analysis of differentially expressed genes

Supplementary Table 1.1 - Biological process

	**Functional annotation term**	**Count**	**%**	***P*-value**
	Translation	30	8.1	2.5E-10
*	Cell adhesion	27	7.3	8.6E-07
*	Homophilic cell adhesion via plasma membrane adhesion molecules	14	3.8	8.6E-06
	Mitophagy in response to mitochondrial depolarization	9	2.4	0.0028
*	Cell-matrix adhesion	7	1.9	0.0029
*	Regulation of oligodendrocyte progenitor proliferation	3	0.8	0.0031
*	Sensory perception of sound	9	2.4	0.0037
*	Positive regulation of cell migration	11	3	0.0039
*	Negative regulation of axon extension	4	1.1	0.0053
*	Axon guidance	9	2.4	0.0058
	Ribosomal small subunit assembly	4	1.1	0.0061
	Positive regulation of fibroblast proliferation	6	1.6	0.0064
	rRNA processing	8	2.2	0.008
	Self-proteolysis	3	0.8	0.0085
*	Transmembrane receptor protein tyrosine kinase signaling pathway	7	1.9	0.0096
	Ribosomal large subunit assembly	4	1.1	0.011
*	Negative regulation of cell migration	7	1.9	0.013
*	Single organismal cell-cell adhesion	7	1.9	0.014
*	Heterophilic adhesion via plasma membrane cell adhesion molecules	5	1.4	0.014
	Cell morphogenesis	6	1.6	0.016
	Proteoglycan biosynthetic process	3	0.8	0.016
*	Extracellular matrix organization	7	1.9	0.017
	Positive regulation of transcription, DNA-templated	19	5.1	0.018
	Negative regulation of transcription, DNA-templated	19	5.1	0.018
*	Roundabout signaling pathway	3	0.8	0.019
*	Ion transport	19	5.1	0.02
	Fibroblast migration	3	0.8	0.022
*	Regulation of cell-cell adhesion	3	0.8	0.022
*	Neuronal action potential	4	1.1	0.023
	Potassium ion transmembrane transport	6	1.6	0.024
	Negative regulation of transcription from RNA polymerase II promoter	22	5.9	0.025
	Protein localization to nucleus	4	1.1	0.025
	Regulation of blood pressure	5	1.4	0.026
	Regulation of heart rate	4	1.1	0.027
	Positive regulation of epithelial cell migration	4	1.1	0.027
*	Potassium ion transport	7	1.9	0.028
*	Positive regulation of phosphatidylinositol 3-kinase signaling	5	1.4	0.029
	Atrioventricular valve morphogenesis	3	0.8	0.029
*	Adult behavior	4	1.1	0.031
	Protein complex assembly	5	1.4	0.032
	Positive regulation of cartilage development	3	0.8	0.033
	Histone H3-K4 methylation	3	0.8	0.033
	Negative regulation of negative chemotaxis	2	0.5	0.036

	Chemorepulsion involved in postnatal olfactory bulb migration	2	0.5	0.036
*	Regulation of ion transmembrane transport	7	1.9	0.036
*	Nervous system development	13	3.5	0.042
*	Dendrite morphogenesis	4	1.1	0.043
	Ribosomal small subunit biogenesis	3	0.8	0.046

Supplementary Table 1.2 - Cellular component

	**Functional annotation term**	**Count**	**%**	***P*-value**
	Ribosome	25	6.8	2.8E-14
	Cytosolic large ribosomal subunit	16	4.3	5.1E-11
	Cytosolic large ribosomal subunit	12	3.2	1.5E-9
	Intracellular ribonucleoprotein complex	25	6.8	2.4E-9
*	Focal adhesion	24	6.5	4.3E-7
	Small ribosomal subunit	7	1.9	9.1E-6
*	Membrane	158	42.7	5.8E-5
	T-tubule	8	2.2	5.8E-5
*	Extracellular matrix	16	4.3	2.2E-4
	Z-disk	10	2.7	3.3E-4
	Extracellular exosome	70	18.9	4.8E-4
	Cytoplasm	145	39.2	7.9E-4
*	Perineuronal net	3	0.8	9.0E-4
	Intracellular	46	12.4	9.8E-4
*	Neuron projection	18	4.9	0.0012
	Mitochondrial respiratory chain complex I	6	1.6	0.0013
*	Postsynaptic membrane	12	3.2	0.0019
*	Cell junction	11	3	0.0029
	Actin cytoskeleton	11	3	0.0029
	Proteinaceous extracellular matrix	14	3.8	0.0039
	Mitochondrion	46	12.4	0.004
	Sarcolemma	8	2.2	0.0047
	Stress fiber	6	1.6	0.0063
	Basement membrane	7	1.9	0.0071
*	Synapse	18	4.9	0.0078
	Histone methyltransferase complex	4	1.1	0.01
	Nuclear body	5	1.4	0.012
	Intercalated disc	4	1.4	0.012
	Cytoskeleton	31	8.4	0.013
*	Neuronal cell body membrane	4	1.1	0.014
	Cell surface	20	5.4	0.015
	Flotillin complex	3	0.8	0.015
*	Adherents junction	5	1.4	0.015
	Apical part of cell	7	1.9	0.016
	Membrane raft	11	3	0.017
	Caveola	6	1.6	0.017
	Methylosome	3	0.8	0.018
	Sarcoplasmic reticulum	5	1.4	0.018
	Transcriptional repressor complex	5	1.4	0.018
	Respiratory chain	5	1.4	0.018
	Mitochondrial inner membrane	14	3.8	0.019
	Sarcoplasmic reticulum membrane	4	1.1	0.02

	Histone deacetylase complex	4	1.1	0.025
*	Postsynaptic density	10	2.7	0.025
	PML body	6	1.6	0.026
	Protein complex	19	5.1	0.028
	Nuclear speck	9	2.4	0.028
*	Axon	13	3.5	0.03
*	Site of polarized growth	2	0.5	0.035
	Basal part of cell	3	0.8	0.035
	Perinuclear region of cytoplasm	20	5.4	0.035
	Basal plasma membrane	4	1.1	0.037
	Costamere	3	0.8	0.043
	Brush border	5	1.4	0.046
	Filopodium	5	1.4	0.046

Supplementary Table 1.3 - Molecular function

	**Functional annotation term**	**Count**	**%**	***P*-value**
	Structural constituent of ribosome	29	7.8	3.2E-14
	Transmembrane receptor protein tyrosine kinase	9	2.4	4.6E-06
	Poly(A) RNA binding	39	10.5	8.4E-5
*	Acting binding	17	4.6	3.8E-0.4
	Calcium ion binding	25	6.8	0.0016
*	Protein binding	96	25.9	0.002
*	Scaffold protein binding	6	1.6	0.0025
	Zinc ion binding	33	8.9	0.0029
	NADH dehydrogenase activity	5	1.4	0.0033
	Metal ion binding	80	21.6	0.0039
	Ion channel activity	9	2.4	0.011
	Potassium channel activity	6	1.6	0.014
	Histone-lysine N-methyltransferase activity	4	1.1	0.02
	Collagen binding	5	1.4	0.021
	Transcription corepressor activity	8	2.2	0.022
*	Identical protein binding	19	5.1	0.031
	Voltage-gated ion channel activity	7	1.9	0.033
*	Protein C-terminus binding	9	2.4	0.034
	Inhibitory extracellular ligand-gated ion channel activity	2	0.5	0.035
	Growth factor binding	4	1.1	0.038
	Histone methyltransferase activity (H3-K4 specific)	3	0.8	0.44

Supplementary Table 1.4 - KEGG pathways

**Pathways**	**Count**	**%**	***P*-value**
Ribosome	29	7.8	2.0E-20
Adherens junctions	7	1.9	0.0031
Circadian entrainment	8	2.2	0.0034
Axon guidance	9	2.4	0.0043
ECM-receptor interaction	7	1.9	0.0082
Focal adhesion	11	3	0.0085
Huntington's disease	10	2.7	0.018

Oxidative phosphorylation	8	2.2	0.021
Alzheimer's disease	9	2.4	0.026
Parkinson's disease	8	2.2	0.032
GABAergic synapse	6	1.6	0.032
Morphine addiction	6	1.6	0.038
Ras signaling pathway	10	2.7	0.042

GO enrichment analysis of 416 DEGs comparing *Cdh2*-mutated and WT littermates, extracted from both the vMB and PFC (n=8 for each strain, four samples per tissue). We focused only on genes with adjusted *p*-values lower than or equal to 0.05 and a fold change higher than or equal to 1.3. Data were retrieved using the DAVID functional annotation tool (https://david.ncifcrf.gov/summary.jsp/). The DAVID Functional Annotation Clustering function uses a Kappa statistic score to measure relationships among the annotation terms based on the degrees of their co-association genes. All statistically significant ($P<0.05$) enriched GO terms in biological process (Supplementary Table 1.1), cellular component (Supplementary Table 1.2) and molecular function (Supplementary Table 1.3) branches are presented. Significantly enriched pathways in KEGG pathway analysis of DEGs are shown in Supplementary Table 1.4. Asterisks denote the most relevant GO terms related to our hypothesis, mainly neuronal functioning, synaptogenesis, cellular adhesion and behavioral features. Count: genes involved in the term. %: percentage (involved genes/total genes). One-sided *p*-values for Modified Fisher's Exact test are defined (the smaller the more enriched). DEGs, differentially expressed genes; GO, Gene Ontology; DAVID, Database for Annotation, Visualization and Integrated Discovery; KEGG, Kyoto Encyclopedia of Genes and Genomes; PFC, prefrontal cortex; vMB, ventral midbrain.

Supplementary Table 2

GO Biological process analysis – ventral midbrain (vMB)

	Functional annotation term	Count	%	*P*-value
	Translation	45	7.9	2.9E-15
	Ribosomal small subunit assembly	7	1.2	1.5E-5
*	Axon guidance	14	2.5	2.2E-4
*	Regulation of cell migration	10	1.8	2.3E-4
	Angiogenesis	18	3.2	3.0E-4
*	Transport	73	12.8	7.3E-4
	Transcription, DNA-template	74	13	0.0011
	Ribosomal small subunit biogenesis	5	0.9	0.0015
*	Cell adhesion	26	4.6	0.0018
	Photoreceptor cell maintenance	6	1.1	0.0043
*	Semaphorin-plexin signaling pathway involved in axon guidance	4	0.7	0.0046
	Regulation of translation	10	1.8	0.0058
	Cytoskeleton-dependent intracellular transport	4	0.7	0.0058
	Activation of JNKK activity	3	0.5	0.007
	rRNA processing	10	1.8	0.0075
*	Nervous system development	20	3.5	0.0075
	Heart morphogenesis	7	1.2	0.0077
	In utero embryonic development	17	3	0.0079
	Histone H3-K4 methylation	4	0.7	0.0086
*	Single organismal cell-cell adhesion	9	1.6	0.0092
	Calcium ion transmembrane transport	8	1.4	0.012
	miRNA mediated inhibition of translation	4	0.7	0.012
*	Regulation of short-term neuronal synaptic plasticity	4	0.7	0.012
	DNA repair	17	3	0.014
*	Homophilic cell adhesion via plasma membrane adhesion molecules	11	1.9	0.014
*	Cell differentiation	33	5.8	0.014
*	Positive regulation of dendrite development	4	0.7	0.014
	Covalent chromatin modification	15	2.6	0.014
	Calcium ion transport	10	1.8	0.015
	Retinal ganglion cell axon guidance	4	0.7	0.016
	Embryonic hemopoiesis	4	0.7	0.018
	Protein acetylation	3	0.5	0.018
*	Proton transport	6	1.1	0.02
	Calcium ion import	4	0.7	0.024
	ATP synthesis coupled proton transport	4	0.7	0.024
	Negative regulation of microtubule depolymerization	4	0.7	0.026
*	Neuron projection extension	4	0.7	0.026
*	Dendrite morphogenesis	5	0.9	0.029
	Positive regulation of fibroblast proliferation	6	1.1	0.032
	Negative chemotaxis	4	0.7	0.033
	Ribosomal large subunit assembly	4	0.7	0.33
*	Cell-cell adhesion	11	1.9	0.034
*	Branchiomotor neuron axon guidance	3	0.5	0.034
	Cardiac conduction	3	0.5	0.034
*	Extracellular matrix organization	8	1.4	0.036
*	Neuron projection development	9	1.6	0.036
	Response to hypoxia	11	1.9	0.037

	Positive regulation of nuclear mRNA poly(A) tail shortening	3	0.5	0.041
*	Roundabout signaling pathway	3	0.5	0.041
	Sprouting angiogenesis	4	0.7	0.043
	Spindle assembly	4	0.7	0.043
*	Synaptic transmission, glutamatergic	4	0.7	0.043
	Regulation of transcription, DNA-templated	76	13.4	0.045
	Negative regulation of angiogenesis	6	1.1	0.046
*	Regulation of cell-cell adhesion	3	0.5	0.047
	Mitochondrial electron transport	3	0.5	0.047
	Positive regulation of transcription from RNA polymerase II	37	6.5	0.049
*	Learning or memory	5	0.9	0.049

GO enrichment analysis of 604 differentially expressed genes comparing *Cdh2*-mutated and WT littermates extracted from the vMB (n=8, four samples in each group). We focused only on genes with adjusted *p*-values lower than or equal to 0.05 and a fold change higher than or equal to 1.3. Data were retrieved using the DAVID functional annotation tool (https://david.ncifcrf.gov/summary.jsp/). All statistically significant ($P<0.05$) enriched GO terms in the biological process domain are presented. Asterisks denote the most relevant GO terms related to our hypothesis, mainly neuronal functioning, synaptogenesis, cellular adhesion and behavioral features. Count: genes involved in the term. %: percentage (involved genes/total genes). One-sided *p*-values for Modified Fisher's Exact test are defined (the smaller, the more enriched). GO, Gene Ontology; DAVID, Database for Annotation, Visualization and Integrated Discovery; vMB, ventral midbrain.

Supplementary Table 3

GO Biological processing analysis – Prefrontal cortex (PFC)

	**Functional annotation term**	**Count**	**%**	***P*-value**
	Translation	26	15.6	1.2E-15
	Ribosomal small subunit assembly	5	3.0	2.1E-5
*	Axon guidance	8	4.8	1.9E-4
	Ribosomal small subunit biogenesis	4	2.4	4.4E-4
	Cytoplasmic translation	4	2.4	0.0025
	Regulation of heart rate	4	2.4	0.0029
	rRNA processing	6	3.6	0.0034
	Ventricular septum development	4	2.4	0.0034
*	Cell adhesion	11	6.6	0.0055
	Maturation of SSU-rRNA	3	1.8	0.007
	Calcium ion import	3	1.8	0.014
	Ribosomal large subunit assembly	3	1.8	0.018
*	Motor neuron axon guidance	3	1.8	0.021
	Fibroblast proliferation	2	1.2	0.031
*	Synapse organization	3	1.8	0.035
*	Cell-substrate junction assembly	2	1.2	0.039
*	Protein retention in Golgi apparatus	2	1.2	0.039
	Negative regulation of chemokine-mediated signaling pathway	2	1.2	0.039
	Mitochondrial DNA repair	2	1.2	0.047
	Positive regulation of sodium ion transporter activity	2	1.2	0.047
	Regulation of hair cycle	2	1.2	0.047
	Ribosomal subunit export from nucleus	2	1.2	0.047

GO enrichment analysis of 181 differentially expressed genes comparing *Cdh2*-mutated and WT littermates extracted from the PFC (n=8, four samples in each group). We focused only on genes with adjusted *p*-values lower than or equal to 0.05 and a fold change higher than or equal to 1.3. Data were retrieved using the DAVID functional annotation tool (https://david.ncifcrf.gov/summary.jsp/). All statistically significant (P<0.05) enriched GO terms in the biological process are presented. Asterisks denote the most relevant GO terms related to our hypothesis, mainly neuronal functioning, synaptogenesis, cellular adhesion and behavioral features. Count: genes involved in the term. %: percentage (involved genes/total genes). One-sided *p*-values for Modified Fisher's Exact test are defined (the smaller, the more enriched). GO, Gene Ontology; DAVID, Database for Annotation, Visualization and Integrated Discovery; PFC, prefrontal cortex.

Supplementary Table 4

Differentially expressed genes in *Cdh2* knock-in mice

Gene symbol	OMIM Number	Adjusted *P*-value	FC
**Adult behavior genes:**			
ZFHX2	* 617828	1.06E-05	-1.389
UNC79	* 616884	4.72E-03	-1.305
SHANK1	* 604999	2.75E-10	-1.466
ALK	* 105590	9.42E-03	-1.356
**Cell-adhesion genes:**			
DSCAML1	* 611782	2.45E-03	-1.311
ACAN	* 155760	0.026	-1.354
CDH2	*** 114020**	**2.77E-04**	**-1.493**
CDH5	* 601120	3.80E-04	-1.505
CELSR2	* 604265	4.58E-07	-1.300
CELSR3	* 604264	7.74E-07	-1.334
COL7A1	* 120120	0.049	-1.549
CNTNAP5	* 610519	0.011	-1.496
DCHS1	* 603057	4.62E-05	-1.498
DST	* 113810	1.70E-13	-1.395
FN1	* 135600	1.54E-07	-1.786
IGSF9B	* 613773	5.69E-04	-1.758
ITGA1	* 192968	6.85E-04	-1.854
LAMC3	* 604348	6.29E-04	-1.890
LAMC2	* 150292	0.043	-1.356
MPDZ	* 603785	3.40E-05	-1.392
NID1	* 131390	1.22E-03	-1.411
NINJ1	* 602062	8.31E-05	1.347
PODXL	* 603632	3.97E-04	-1.501
PCDH15	* 605514	9.22E-04	-1.491
SDK2	* 607217	3.24E-03	-1.598
TNR	* 601995	4.34E-05	-1.652
TENM2	* 610119	5.27E-05	-1.457
TENM3	* 610083	0.011	-1.449
VCL	* 193065	5.08E-05	-1.334
**Neuronal action potential genes:**			
ANK3	* 600465	2.93E-06	-1.366
CACNA1I	* 608230	1.82E-05	-1.370
KCNMA1	* 600150	9.02E-03	-1.420
KCNA2	* 176262	0.028	-1.442

**Axon guidance:**			
ANK3	* 600465	2.93E-06	-1.366
DCC	* 120470	3.31E-04	-1.738
EFNA5	* 601535	0.026	-1.382
KIF26B	* 614026	0.026	-1.338
PLXNA4	* 604280	1.43E-05	-1.443
ROBO1	* 602430	2.42E-05	-1.312
ROBO2	* 602431	9.70E-04	-1.330
SLIT2	* 603746	4.16E-04	-1.405
TENM2	* 610119	5.27E-05	-1.457
**Regulation of cell-cell adhesion:**			
CELSR2	* 604265	4.58E-07	-1.300
EFNA5	* 601535	0.026	-1.382
PODXL	* 603632	3.97E-04	-1.501
**Nervous system development:**			
DSCAML1	* 611782	2.45E-03	-1.311
SHANK1	* 604999	2.75E-10	-1.466
ALK	* 105590	9.42E-03	-1.356
CIT	* 605629	3.31E-05	-1.309
DCC	* 120470	3.31E-04	-1.738
EFNA5	* 601535	0.026	-1.382
IGSF9B	* 613773	5.69E-04	-1.758
NAV1	* 611628	1.53E-05	-1.313
NAV2	* 607026	3.24E-13	-1.466
PLXNA4	* 604280	1.43E-05	-1.443
ROBO1	* 602430	2.42E-05	-1.312
ROBO2	* 602431	9.70E-04	-1.330
SLIT2	* 603746	4.16E-04	-1.405

Significance and fold change of differentially expressed genes in the *Cdh2* knock-in mice model. The DAVID Functional Annotation Clustering function uses a Kappa statistic score to measure relationships among the annotation terms based on the degrees of their co-association genes. A few Significantly relevant GO terms' genes are presented (listed in Supplementary Table 1). One-sided *p*-values for Modified Fisher's Exact test are defined. FC, fold change. Note that the *Cdh2* expression is down-regulated, as one of the differentially expressed cell-adhesion term genes.

Supplementary Table 5

Primers and oligos

Gene/oligo	primer	Sequence
CDH2 - Human	Forward	5'-ATAGTTTACACAGCTGTGCC-3'
	Reverse	5'-GACCTACAAGTGAGCTCTAC-3'
Cdh2 - Mouse	Forward	5'-TCCAGTGAATAGTGCAACCC-3'
	Reverse	5'-CAAGAGACGACACAGGAAAG-3'
sgRNA		AGCACAGTGGAGCTCTACAA
ssODN*		AAAGGGTCCTCTGGAGTTTTCTGGCAAGTTGATTGGC GGGATGACCCAGTCTCTCTTCTGgCgTTGTAGAGCTC CACTGTaCTTGCAAGTTGTCTAGGGAATACTATTTCT TCAATTTCATGTGGTTCCTTTGCATTAAA
TH	Forward	5'-TGTTGGCTGACCGCACAT-3'
	Reverse	5'-GCCCCCAGAGATGCAAGT-3'
DAT	Forward	5'-ACTTCAGGGAAGGTGGTGTGGAT-3'
	Reverse	5'-GTAGAAGTCCACACTGAGGTATGC-3'
Mouse 18S rRNA	Forward	5'-GTAACCCGTTGAACCCCATT-3'
	Reverse	5'-CCATCCAATCGGTAGTAGCG-3'

Oligos used to generate knock-in mice. Primers used for Sanger sequencing and RT-qPCR analysis. * Mutation site and synonymous replacement of specific PAM (protospacer adjacent motif) nucleotides are denoted with a lower case. ssODN, single-stranded donor oligonucleotides; TH, tyrosine hydroxylase; DAT, dopamine transporter.

Supplementary Figure 1

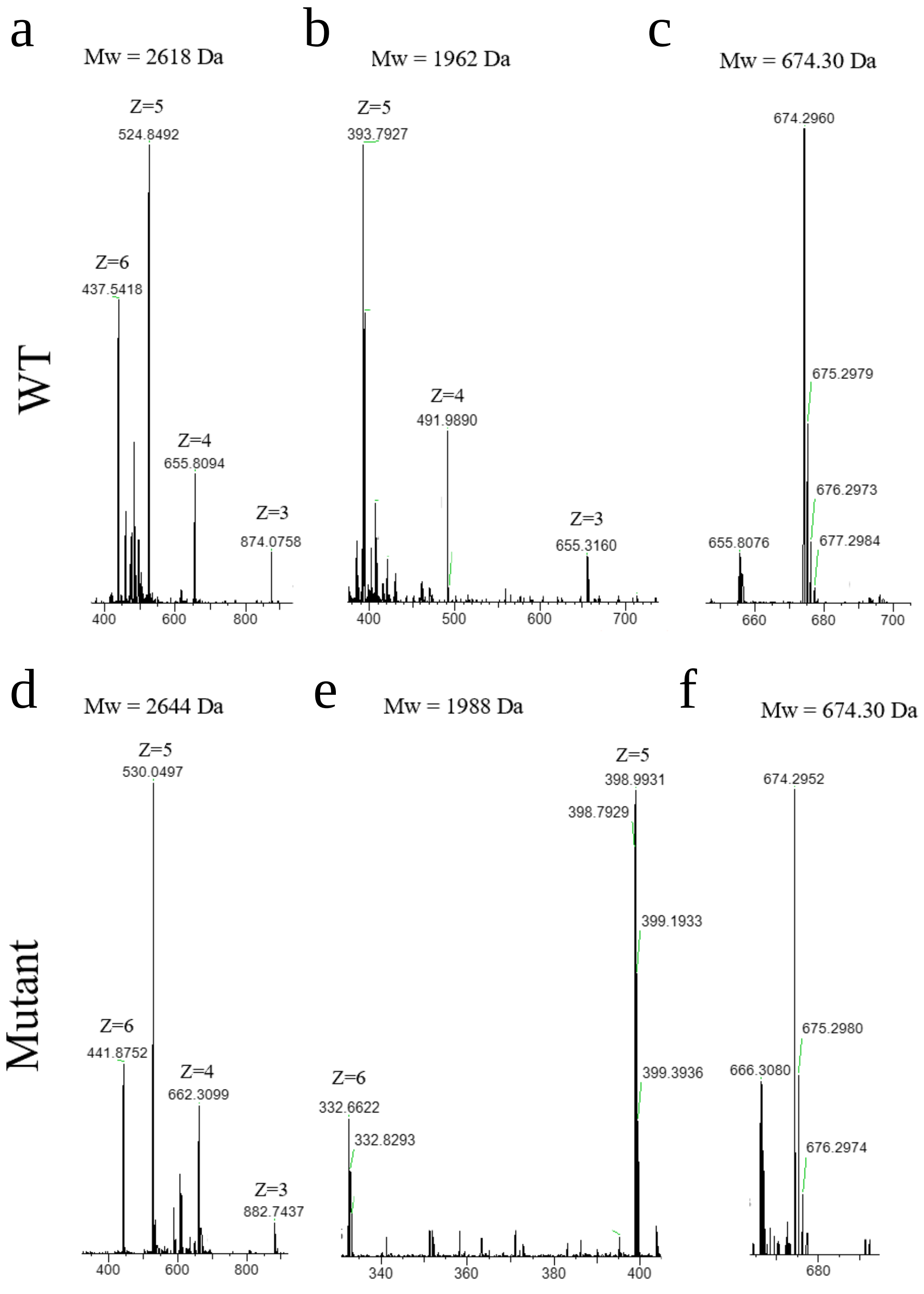

Supplementary Figure 1; MS spectra of peptides following furin protease digestion assay

To test whether the p.H150Y mutation interferes with protein processing, we performed a biochemical peptide cleavage assay using furin protease, the prototypical proprotein convertase; 22 amino-acid WT and mutant peptides were synthesized (GL Biochem, Shanghai), harboring the RXK/R-R recognition and cleavage motif, conjugated with FITC and biotin at their N and C-terminus, respectively (WT:FITC-SK***H***SGHL<u>QRQKR/</u>DW-K-biotin, Mutant: FITC-SK***Y***SGHL<u>QRQKR/</u>DW-K-biotin, Fig. 2c). Following digestion with furin (Fig. 2d), peptides were cleaved into two fragments based on the recognition preference of the protease. Digests were then subjected to liquid chromatography-mass spectrometry (LC-MS) analysis. Demonstrated are MS spectra of WT and mutant peptides; (a-c) WT peptides; (a) full-length (Rt~5 min) (b) large-fragment (FITC-SK***H***SGHL<u>QRQKR)</u> (c) small-fragment peptide (Rt~4.3 min, DW-K-biotin). (d-f) Mutant peptides. (d) full-length (Rt~5 min) (e) large-fragment (FITC-SK***Y***SGHL<u>QRQKR</u>) (f) small-fragment peptide (Rt~4.3 min, DW-K-biotin). Analyzed were performed using a Thermo Scientific Dionex Ultimate 3000 UPLC system coupled to a Q Exactive Focus mass spectrometer (Thermo Scientific, San Jose, USA). Electrospray analysis was performed in the positive ionization mode using a spray voltage of 2.5-3.5 kV, the tune settings for the MS used an S-lens setting of 50. A full scan range of 300–2000 m/z was used at a resolution of 70,000. X-axis: m/z; Rt, retention time.

Supplementary Figure 2

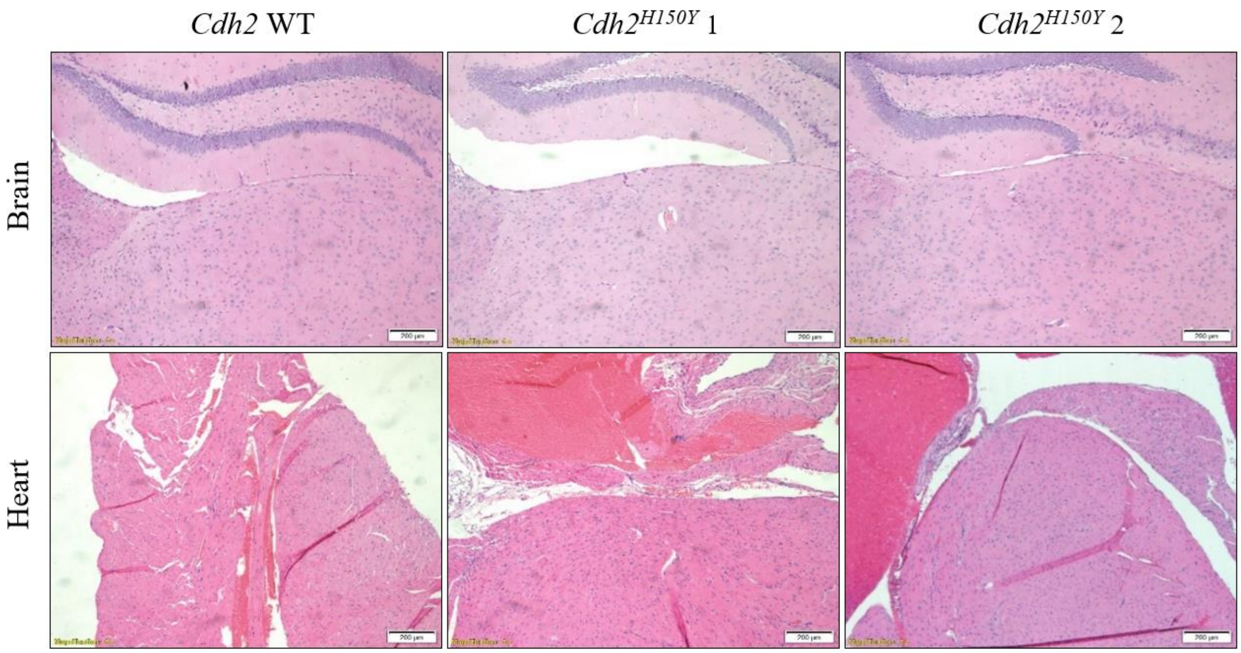

Supplementary Figure 2; Knock-in mice phenotypic evaluation and histological assessment

Thorough pathological studies of homozygous 10-week-old mutant mice, with a special focus on the brain, demonstrated neither anatomical nor histological abnormalities in any of the tissues examined. Presented are brain and heart tissue samples. Other tissues examined: lung, thymus, kidney, liver, spleen, small intestine, large intestine, stomach, eyes, skin, striated muscle, urinary bladder, duct. deference, testis, vesicular gland, thyroid, salivary gland, harderian gland, lymph node, pancreas, cartilage, bone and bone marrow. Slide preparation: Paraffin blocks were sectioned at ~4 microns thickness. Sections were put on glass slides and stained with Hematoxylin & Eosin (H&E). pictures were taken using Olympus microscope (BX60. serial NO. 7D04032) at an objective magnification of X1.25 and X4. H&E stained sections were examined by one pathologist (www.patho-logica.com) and scored by a semi-quantitative scoring system for the presence of pathological changes (histological assessment was preformed once, with n=3: 1 WT mice, 1 mutant mice strain 1- *Cdh2*$^{H150Y(1)}$, 1 mutant mice strain 2- *Cdh2*$^{H150Y(2)}$). A complete histopathological report is available upon request (study number: BGD-848-HIS at patho-logica.com).
