# Supplementary material for: CDH2 mutation affecting N-cadherin function causes attention-deficit hyperactivity disorder in humans and mice
Source: Nat Commun. 2021 Oct 26;12:6187. doi: 10.1038/s41467-021-26426-1 (PMC8548587; doi:10.1038/s41467-021-26426-1)
Supplement: Supplementary file 3 — Description of Additional Supplementary Files [file 41467_2021_26426_MOESM3_ESM.pdf]

Description of Additional Supplementary Files

Supplementary Data 1: DESeq2 RNA-sequencing data

Transcriptome analysis of micro-dissected ventral midbrain (vMB) and prefrontal cortex (PFC) brain tissues of 13-week-old male WT and homozygous *Cdh2*^{H150Y} mice was performed (n=16, four samples per tissue per genotype). After applying adjusted *P*-values to the RNA-seq data, hierarchical clustering (Fig. 8a, b) identified 181 differentially expressed genes (DEGs) within the PFC, of which 99 were downregulated and 82 were upregulated in the mutants. Hierarchical clustering of the vMB identified 604 DEGs, with 383 and 221 genes down and upregulated in the mutants, respectively. Raw reads were obtained by sequencing on Illumina's NextSeq 500, single end 60; They were pre-processed by trimming Illumina adapters (GATCGGAAGAGCACACGTCTGAACTCCAGTCAC) and also by trimming edges of reads with quality below 10 using Cutadapt v1.8.3. Reads with lengths below 40 were discarded. Next, reads that contained more than 50% A or 50% T were also discarded. The processed reads were mapped to the human genome, GRCh38, using TopHat v2.0.10 and then the number of reads that were mapped to each gene were calculated using HTSeq-count, v0.6.1p1. The gene annotation used for the counting was Ensemble annotation for GRCh38, release 83. Differential expression was assayed using Deseq2, comparing samples that were *Cdh2*-mutated using CRISPR/Cas9 versus WT. Genes demonstrating absolute log fold change greater than or equal to 1.3, as well as adjusted p-value lower than or equal to 0.05, were considered as differentially expressed. Adjusted p-values were taken from Deseq correction for multiple testing, which is based on Benjamin-Hochberg FDR. Genes that were differentially expressed in both cases were considered for further analysis.
