# Supplementary material for: CDH2 mutation affecting N-cadherin function causes attention-deficit hyperactivity disorder in humans and mice
Source: Nat Commun. 2021 Oct 26;12:6187. doi: 10.1038/s41467-021-26426-1 (PMC8548587; doi:10.1038/s41467-021-26426-1)
Supplement: Supplementary file 5 — Reporting Summary [file 41467_2021_26426_MOESM5_ESM.pdf]

Corresponding author(s): Ohad S. Birk

Last updated by author(s): Aug 23, 2021

Reporting Summary

Nature Research wishes to improve the reproducibility of the work that we publish. This form provides structure for consistency and transparency in reporting. For further information on Nature Research policies, see our Editorial Policies and the Editorial Policy Checklist.

Statistics

For all statistical analyses, confirm that the following items are present in the figure legend, table legend, main text, or Methods section.

n/a	Confirmed	
☐	☒	The exact sample size (*n*) for each experimental group/condition, given as a discrete number and unit of measurement
☐	☒	A statement on whether measurements were taken from distinct samples or whether the same sample was measured repeatedly
☐	☒	The statistical test(s) used AND whether they are one- or two-sided<br>*Only common tests should be described solely by name; describe more complex techniques in the Methods section.*
☐	☒	A description of all covariates tested
☒	☐	A description of any assumptions or corrections, such as tests of normality and adjustment for multiple comparisons
☐	☒	A full description of the statistical parameters including central tendency (e.g. means) or other basic estimates (e.g. regression coefficient) AND variation (e.g. standard deviation) or associated estimates of uncertainty (e.g. confidence intervals)
☐	☒	For null hypothesis testing, the test statistic (e.g. *F*, *t*, *r*) with confidence intervals, effect sizes, degrees of freedom and *P* value noted<br>*Give P values as exact values whenever suitable.*
☒	☐	For Bayesian analysis, information on the choice of priors and Markov chain Monte Carlo settings
☒	☐	For hierarchical and complex designs, identification of the appropriate level for tests and full reporting of outcomes
☒	☐	Estimates of effect sizes (e.g. Cohen's *d*, Pearson's *r*), indicating how they were calculated

Our web collection on statistics for biologists contains articles on many of the points above.

Software and code

Policy information about availability of computer code

Data collection

For data collection we used the following:
1. Genome-wide single nucleotide polymorphism (SNP) distributions; llumina's Omni Express bead-chip (>750k/sample); https://genebygene.com/
2. Whole-exome sequencing data; llumina's NovaSeq 6000 platform; https://dna.macrogen.com/
3. Whole-transcriptome RNA-sequencing data; Illumina's HiSeq-2500 platform; https://g-incpm.weizmann.ac.il/units/CrownGenomics/
4. SNP database; http://www.ncbi.nlm.nih.gov/projects/SNP/
5. Mouse Genome Informatics: http://www.informatics.jax.org/
6. PDB, protein data bank; https://www.rcsb.org/

Data analysis

Data analyses were performed utilizing commercially or publicly available software. The following softwares were used to process and analyze data:
1. QIAGEN's Ingenuity variant analysis software: https://digitalinsights.qiagen.com/
2. UCSC genome browser; https://genome.ucsc.edu/
3. IGSR: The International Genome Sample Resource; https://www.internationalgenome.org/
4. GenomAD browser v2.1.1: https://gnomad.broadinstitute.org/
5. HomozygosityMapper: http://www.homozygositymapper.org/
6. Online Mendelian Inheritance in Man (OMIM); http://www.omim.org/
7. Clustal Omega software: http://www.ebi.ac.uk/Tools/msa/clustalo/
8. SnapGene software v5.3: https://www.snapgene.com/

9. Axon™pCLAMP™ 10 Electrophysiology Data Acquisition & Analysis, v10.3.2.1; https://moleculardevices.app.box.com/s/rraipn0bep04avbr2tcufxww6at4ort5/
10. PyMOL molecular graphics v1.2r1; DeLano Scientific LLC; https://sourceforge.net/projects/pymol/
11. SWISS-MODEL server: https://swissmodel.expasy.org/
12. HPEPDOCK server v2021-06-05; https://omictools.com/hpepdock-tool/
13. NIS-Elements vAR 5.21.03 imaging software; https://www.microscope.healthcare.nikon.com/products/software/nis-elements/nis-elements-advanced-research/
14. OriginPro2020; https://www.originlab.com/2020/
15. DAVID, The Database for Annotation, Visualization and Integrated Discovery v6.8; https://david.ncifcrf.gov/
16. Ethovision v11 video tracking software; https://www.noldus.com/ethovision-xt/
17. Heatmapper; http://www.heatmapper.ca/
18. SPSS v18.0 software package

For manuscripts utilizing custom algorithms or software that are central to the research but not yet described in published literature, software must be made available to editors and reviewers. We strongly encourage code deposition in a community repository (e.g. GitHub). See the Nature Research guidelines for submitting code & software for further information.

Data

Policy information about availability of data

All manuscripts must include a data availability statement. This statement should provide the following information, where applicable:

- Accession codes, unique identifiers, or web links for publicly available datasets
- A list of figures that have associated raw data
- A description of any restrictions on data availability

Whole-exome sequencing data are available from the corresponding author upon request (due to medical confidentiality and regulations, WES information cannot be deposited to an open repository). The raw and processed mouse RNA-sequencing data generated in this study have been deposited in the Gene Expression Omnibus database under accession code XXX. Regarding structural data, CDH2 protein and furin protease modeling was predicted using the SWISS-MODEL server (at PDB ID 6E6B and PDB ID 4Z2A, respectively). Other data generated in this study are provided in the Supplementary Information, and all other data that support the findings of this study are provided in Source Data file.

Field-specific reporting

Please select the one below that is the best fit for your research. If you are not sure, read the appropriate sections before making your selection.

☒ Life sciences ☐ Behavioural & social sciences ☐ Ecological, evolutionary & environmental sciences

For a reference copy of the document with all sections, see nature.com/documents/nr-reporting-summary-flat.pdf

Life sciences study design

All studies must disclose on these points even when the disclosure is negative.

Sample size	Genetic analysis: all family members who agreed to participate in the study were recruited for genome-wide linkage analysis, and one affected individual was analyzed for whole-exome sequencing. Mice behavioral experiments: no sample size calculation was performed. Instead, the maximum number of male homozygous WT and mutant littermates, born on the same 1-2 days interval to heterozygous parents, where used for behavioral and cognitive evaluation (n=18 and n=30, respectively). For all in-vitro experiments: sample size was chosen such that each condition was sampled from at least 3 independent sources (mice, neuronal cultures, brain slices), each producing >=3 products (slices, cover slips etc). Each cover slip was sampled once. Each sampling (image) contained multiple measurable units (synapses, cells), numbers as indicated for each experiment. .
Data exclusions	Grubbs's test was used to identify outliers in otherwise normally distributed datasets. Experiments that were deemed to have failed on technical grounds (no responses recorded or similar) were excluded in their entirety.
Replication	Statistical analysis was performed on experiments repeated independently at least 3 times (acquired from at least 3 independent cultures on different days; Immuno-cytochemistry of hippocampal cultures, measuring vesicle cycling using sypHy, synapse width analyses, frequency of spontaneous synaptic release, Schaffer collateral frequency facilitation, pre-synaptic cytoplasmic calcium measurements, real-time quantitative PCR, Tyrosine hydroxylase immunofluorescence visualization). Mice behavioral experiments were conducted twice, on two different knock-in strains, six months apart. In-vitro protein cleavage assays was performed independently 4 times. Similarity of results across similar experimental conditions was tested to assess reproducibility.
Randomization	Experimental design was not random; comparisons were performed in all experiments between data obtained from WT and knock-in mice. Genetic analysis was preformed on a single affected individual.

Blinding	Experiments did not follow a blind design. However, all peptide cleavage assays, mice behavioral evaluation studies, immunofluorescence image analyses, immunocytochemistry synaptic vesicle experiments and dopamine levels measurements were performed by a researcher who was blind to the experimental conditions of the specimens. When applicable, the choice of regions of interest for analysis was performed on images that do not include the measured quantity, or from images in series, in which the measured quantity is not evident (for example, final values at completion of sypHy experiments). Behavioral experiments involving tracking and recording (mean startle amplitude, open-field apparatus test, etc.) were calculated in a fully computerized, blinded and unbiased measurement.

Reporting for specific materials, systems and methods

We require information from authors about some types of materials, experimental systems and methods used in many studies. Here, indicate whether each material, system or method listed is relevant to your study. If you are not sure if a list item applies to your research, read the appropriate section before selecting a response.

Materials & experimental systems

n/a	Involved in the study
☐	☒ Antibodies
☒	☐ Eukaryotic cell lines
☒	☐ Palaeontology and archaeology
☐	☒ Animals and other organisms
☐	☒ Human research participants
☒	☐ Clinical data
☒	☐ Dual use research of concern

Methods

n/a	Involved in the study
☒	☐ ChIP-seq
☒	☐ Flow cytometry
☒	☐ MRI-based neuroimaging

Antibodies

Antibodies used	1. Rabbit polyclonal anti-Synaptobrevin-2 (Synaptic Systems, Cat. No. 104 202)<br>2. Goat polyclonal anti-Vesicular glutamate transporter-1 (vGlut I, Synaptic Systems, Cat. No. 135 307)<br>3. Mouse monoclonal anti-Glutamic acid decarboxylase-6 (GAD-6, developed by D.I. Gottlieb, obtained from the Developmental Studies Hybridoma Bank, DSHB)<br>4. Donkey anti-mouse IgG labeled with NorthernLights 637 (R&D Systems, Cat. No. NL008)<br>5. Donkey anti-rabbit IgG labeled with NorthernLights 557 (R&D Systems, Cat. No. NL004)<br>6. Donkey anti-goat IgG labeled with AlexaFluor 647 (Abcam 150135)<br>7. Mouse anti-Tyrosine hydroxylase (Santa Cruz Bio, sc-25269)<br>8. Donkey anti-mouse with AlexaFluor 488 (Jackson, ImmunoResearch, Code 715-225-150)
Validation	1. https://www.sysy.com/products/s-brevin2/facts-104202.php<br>2. https://www.sysy.com/products/vglut1/facts-135307.php<br>3. https://www.dshb.biology.uiowa.edu/GAD-6<br>4. https://www.rndsystems.com/products/donkey-anti-mouse-igg-northernlights-nl637-conjugated-antibody_nl008<br>5. https://www.rndsystems.com/products/donkey-anti-rabbit-igg-northernlights-nl557-conjugated-antibody_nl004<br>6. https://www.abcam.com/donkey-goat-igg-hl-alexa-fluor-647-preadsorbed-ab150135.html<br>7. https://www.scbt.com/p/th-antibody-f-11<br>8. https://www.jacksonimmuno.com/catalog/products/715-225-150

Animals and other organisms

Policy information about studies involving animals; ARRIVE guidelines recommended for reporting animal research

Laboratory animals	Mice: male 10 to 14-week-old wild-type and CRISPR/Cas9-mutated knock-in C57BL/6JRcc mice were used. The colony was generated and maintained in the animal facility of the Ben-Gurion University of the Negev on a 12:12 hours light/dark schedule with food and water provided ad libitum, at temperature of 20-24°C with 30-70% humidity. For cultures: pups of age P0-P2 of either sex were used. For slice recordings: mice of age P18-P21 days were used.
Wild animals	The study did not involve wild animals.
Field-collected samples	The study did not involve samples collected from the field.
Ethics oversight	The study protocol was approved by the animal care and use committee of the Ben-Gurion University of the Negev (IL-34-05-2019). Behavioral tests were conducted in an SPF-certified examination room at HaddasaBrainLabs (http://brainlabs.org.il). The experimental procedure was according to the ARRIVE guidelines and NIH approval number: OPRR-A01-5011. All experiments were

approved by the Hebrew University Ethics Committee on Animal Care and Use (Applications MD-14-14015-4; MD-16-14679-4). As an AAALAC-accredited Institute, the Hebrew University Ethics Committee follows the NRC Guide for the Care and Use of Laboratory Animals.

Note that full information on the approval of the study protocol must also be provided in the manuscript.

Human research participants

Policy information about studies involving human research participants

Population characteristics	Nine Individuals of consanguineous Bedouin kindred were studied. Three siblings presented with severe attention-deficit hyperactivity disorder diagnosed as of early childhood. All other participants were normal in terms of hyperactivity, intellect and general health.
Recruitment	All available family members who agreed to participate in the study were recruited, with no self-selection bias.
Ethics oversight	DNA samples were obtained following informed consent and approval of the Soroka University Medical Center Internal Review Board (IRB)

Note that full information on the approval of the study protocol must also be provided in the manuscript.
